# Comparison of effect of wobble board training with and without cognitive intervention on balance, ankle proprioception and jump landing kinetic parameters of men with chronic ankle instability: a randomized control trial

**DOI:** 10.1186/s12891-022-05706-x

**Published:** 2022-09-30

**Authors:** Abed Taghavi Asl, Seyed Sadredin Shojaedin, Malihe Hadadnezhad

**Affiliations:** Department of Biomechanics and Sports Injuries, Faculty of Physical Education and Sports Sciences, Kharazmi University, Tehran, Iran

**Keywords:** Balance, Proprioception, Chronic ankle instability, Wobble board training, Cognitive intervention

## Abstract

**Background:**

The aim of the present study was to compare the effect of a wobble board training (WBT) course with and without cognitive intervention (CI) on balance, ankle proprioception and jump-landing kinetic parameters in active men with chronic ankle instability (CAI).

**Methods:**

Twenty-one active men in the age range of 18–25 years with CAI were randomly divided into three groups of WBT for five weeks as follows: with CI (*n* = 7); without CI (*n* = 7); control (*n* = 7). In this study, balance was evaluated by Y-Balance Test and Balance Error Scoring System, proprioception was evaluated as active and passive replication of 15-degree inversion angle and Maximum inversion angle minus 5-degrees, and single-leg jump-landing test was used for measurement of kinetic variables as pre-test and post-test in similar conditions.

**Results:**

Post-test of both groups of WBT with and without CI showed a significant improvement in all variables compared to pre-test (*p* ≤ 0.05); however, in post-test, there was no significant difference in any of the variables between WBT groups with and without CI (*p* ≥ 0.05).

**Conclusions:**

A course of WBT with and without CI leads to a significant improvement in balance, proprioception and jump-landing kinetic parameters, including time to stabilization and center of pressure displacement in active men with CAI. Although the WBT group with CI showed a greater improvement in mean than WBT group without CI, but the difference was not significant in any of the variables.

**Trial registration:**

Retrospectively Registered. irict.ir Identifier: IRCT20200617047818N1 on 05–02-2021.

## Background

The human ankle joint is one of the most vulnerable joints in the body, and the most common type of its injury is anterior talofibular ligament sprain with a reported prevalence of 42–72% [1]. This injury can hurt external ligaments, muscles and nerves of ankle, and damage to anterior talofibular and calcaneofibular ligaments is common in this injury that leads to the development of chronic ankle instability (CAI) due to mechanical and functional factors [2]. Following the initial sprain, 32% of these people develop CAI, which is a frequent complication characterized by symptoms such as giving way, re-sprain and instability. These symptoms limit sports activities and affect daily life for several years [3]. The CAI may be caused by factors such as defective proprioception, neuromuscular control, balance, and strength, or because of mechanical factors such as disorder in one or more joints of the ankle complex [4]. Ankle ligament injuries can be satisfactorily improved with physical rehabilitation, ligament rehabilitation, and non-surgical treatment that do not lead to CAI and do not experience recurrent sprains. This treatment has been mentioned in many articles as a successful treatment [5, 6]. However, half of these people do not seek professional rehabilitation techniques [7].

Posture control in people requires the integration of optic, vestibular, and somatosensory nerve inputs. Somatosensory inputs are a combination of skin, joint, and tendon receptors. The information collected from these three sources is processed by the central nervous system and used for motion control commands. Defects in any of these receptors can lead to defective posture control and balance, which is commonly observed in people with CAI [8, 9].

The foot and ankle play an essential role in reducing shocks during foot contact with the ground, as well as decreasing the speed of incoming loads to prevent ligament rupture. Numerous studies have shown that people with CAI have a higher center of pressure (COP) distribution and lateral pressure while walking [10]. Some other studies have shown that people with CAI have higher inversion (up to 6–7 degrees) while walking before and after the initial contact. Increased inversion may lead to lateral deviation of pressure center, increased lateral pressure of the ankle, and decreased response rate to these loads during the stance phase [11]. On the other hand, Proprioception information from joint and muscle receptors plays an important role in motor control; meanwhile, proprioception information from ankle is significant, which is disrupted by ankle sprain [8]. In general, the proprioception system helps recognize the position and movements of human body by obtaining various information from the body. On the other hand, due to damage to receptors of ankle ligaments and peroneal nerve after initial sprain, neuromuscular function undergoes changes impairing kinaesthesia and sense of force that are prevalent in those with CAI [9] Ankle sprains may occur due to improper foot position before and during contact. Improper foot position may be due to loss of sensory inputs from the mechanoreceptors, increased inversion, and lateral deviation of the center of pressure [7].

Many daily activities require more than one task at a time, which includes dual tasks, which are two motor tasks or one motor task and one cognitive task at the same time [10]. There is a stable relationship between motor function and attention focus that indicates that in order to achieve proper performance in a superficial motor task, attention is needed that this relationship is examined using dual tasks. Therefore, simultaneous motor and cognitive tasks are performed to understand the interplay between the two tasks [11]. It is believed that individuals have limited processing capacity and that each task requires a portion of the overall processing capacity. A degree of attention demand may help motor function optimally, but when these demands exceed processing capacity, motor or cognitive function decreases [12]. In real life, when people with ligament injuries receive multiple tasks simultaneously, it may increase the processing demand. These injured persons have a lower ability to process demands; therefore, the movement pattern can change and lead to subsequent injury [13]. Therefore, given the importance of processing demand and its relationship with the deterioration of motor patterns and eventual musculoskeletal injury, this processing demand can affect rehabilitation protocols and it is one of the main factors meant to restore the function of injured people and prevent re-injury [14].

Wobble board is a simple and inexpensive tool that is widely used to improve balance and control posture [15]. In addition to improving balance quickly and with long-lasting durability, this instrument also reduces ankle sprain injury by up to 50% [16]. Furthermore, wobble board training (WBT) has been reported to assist with reeducation of sensorimotor system by improving mechanoreceptor function and restoring the normal neuromuscular feedback loop [17]. In this regard, Canning et al. (2008) believe that dual tasks should be performed simultaneously to improve and affect complex tasks and activities of daily living [18]. Besides, motor tasks are influenced by adding a simultaneous cognitive task and the ability to perform dual tasks at the same time increases with the repetition of dual task exercises [19].

Many training protocols, including resistance and balance protocols, have been proposed to improve this injury. On the other hand, reviewing literature in this regard has shown that the inclusion of a cognitive intervention (CI) with WBT may be more effective [3]; however, they have not been studied simultaneously in a single research. Therefore, in the present study, we aimed to compare the effect of a WBT course with and without CI on balance, ankle proprioception and some jump-landing kinetic parameters in active men with CAI.

## Methods

### Design

The present study is a randomized controlled trial with three single-blinded groups. In this research, the assessor is blind and does not know how the subjects are assigned to the groups and what treatment intervention each group receives. The method of the present study is experimental and it has been approved by ethics committee of Iranian Institute of Physical Education and Sports Sciences and has also been registered in Iranian Registry of Clinical Trials (IRCT) on 2021–02-05 with code number IRCT20200617047818N1. In this investigation, proprioception, balance, and jump-landing kinetics were considered as dependent variables, while the independent variable was CON, WBT, WBT with CI and time.

### Sample size and randomization

The present study involves active men in the age range of 18–25 years with CAI. Athletes with CAI were identified from several sports clubs using Foot & Ankle Disability Index (FADI) score as well as Foot & Ankle Disability Index-Sports (FADI-S) module and diagnosed by a physician. Inclusion criteria were as follows: active men 18–25 years old, BMI of 18–25 [20], a history of at least two reports of giving way in the last 6 months, and the occurrence of the last injury more than 6 weeks before the start of the study, ability to bear weight on an injured limb (eventually having a slight discomfort), history of at least one significant lateral ankle sprain on one side causing pain and decreasing function for more than a day [21, 22]. Exclusion criteria were a history vestibular or neuro-musculoskeletal disorders, history of lower limb surgery, lower limb injury for at least 6 months prior to the study, consumption of medications affecting balance such as Betahistine, hypertension drugs and sedatives, having musculoskeletal system abnormalities in lower extremities such as flat foot, genu varum, physiotherapy or participation in physical rehabilitation over the last three months. FADI score < 90% and FADI-S < 80% score in subjects were indicative of CAI [23].

The G*Power (Version 3.1.6) was used to determine the sample size. The calculation was based on the F test, with an alpha level of 0.05. The type II error rate was at 80% power and the effect size of 0.4 of the primary outcome variable from similar previous studies [17, 24]. The appropriate sample size for this study was 21 participants whose demographic information is given in Table 1. By simple random sampling, the subjects were individually assigned to control (CON) and intervention groups using a web application (randomizer.org). A member of the research team who was not involved in the selection of samples determined the random allocation sequence using the computer program. Sealed non-transparent envelopes with respective random sequences were used to conceal the allocation, and the subjects were randomly divided to three groups: WBT with CI (*n* = 7); WBT without CI (*n* = 7) and CON (*n* = 7). All the participants in the experimental groups (WBT with CI and WBT) received the intervention and the control group didn’t received intervention during this period of time.

**Table 1:** Anthropometric Variables of Participants between Groups (mean ± SD)

Variables	WBT with CI (*n* = 7)	WBT (*n* = 7)	CON (*n* = 7)	*P*-Value
Age (years)	22.42 ± 2.22	23.14 ± 1.34	22.42 ± 2.43	0.758
Weight (kg)	72.74 ± 3.84	74.57 ± 4.34	76.41 ± 3.51	0.242
Height (cm)	177.57 ± 5.38	174.43 ± 6.43	176.57 ± 5.85	0.603
BMI (kg/m^2^)	21.57 ± 1.01	22.29 ± 0.72	22.33 ± 0.72	0.186

*Note*: *WBT with CI *Wobble Board Training Group with cognitive intervention, *WBT *Wobble Board Training Group, *CON *Control Group

### Exercise protocol

#### Wobble board training group

In the present study, the training protocol of Linens et al. (2016) was used. In this protocol, a CANDO MVP Balance System model board (USA) was employed as a 30-inch circular platform with domes of various dimensions that were wrapped around the bottom of the board to increase or decrease the intensity of exercises. In each session, individuals were asked to perform the following exercises in the three positions of standing on both feet, standing on non-involved foot, and standing on the involved foot on wobble board near the wall. The exercises consisted of 5 sets of 40 s clockwise and counterclockwise rotation each (change of rotation direction every 10 s) with a 60-s break between each set. The subjects could keep their fingers in contact with the wall for more stability. The exercises consisted of 5 levels, and each level increased by half an inch (the elevation range of domes was 1–3 inches). The subjects started their exercises from the lowest level (i.e. the lowest height of the dome) and progressed according to the report of feeling comfortable, as well as the ability of the participant to perform smooth circular rotations in both directions and change uniform directions from clockwise to counterclockwise mode and vice versa. The participants performed these exercises 3 times a week, each session lasting 50 min with warm-up and head exercise for 5 weeks [17].

#### Wobble board training with cognitive intervention group

In this study, the CI of Shiravi et al. (2016) was also used. Subjects in this group were asked to start counting down 7 out of a random number between 200 and 300 in their minds in addition to WBT described in the previous section. Individuals were also requested to be as accurate as possible in performing this CI and to announce the number they had reached at the end. Due to the fact that the subjects are on the Wobble board, if they use verbal or motor interventions, it may upset the balance. Therefore, this CI has been used to prevent any movement disorder in the body [25].

### Control

During this period, the subjects in the CON group performed their normal daily activities and sports in their respective fields and did not receive any of the protocols.

### Protocol of the test

#### Proprioception

**Fig. 1:**
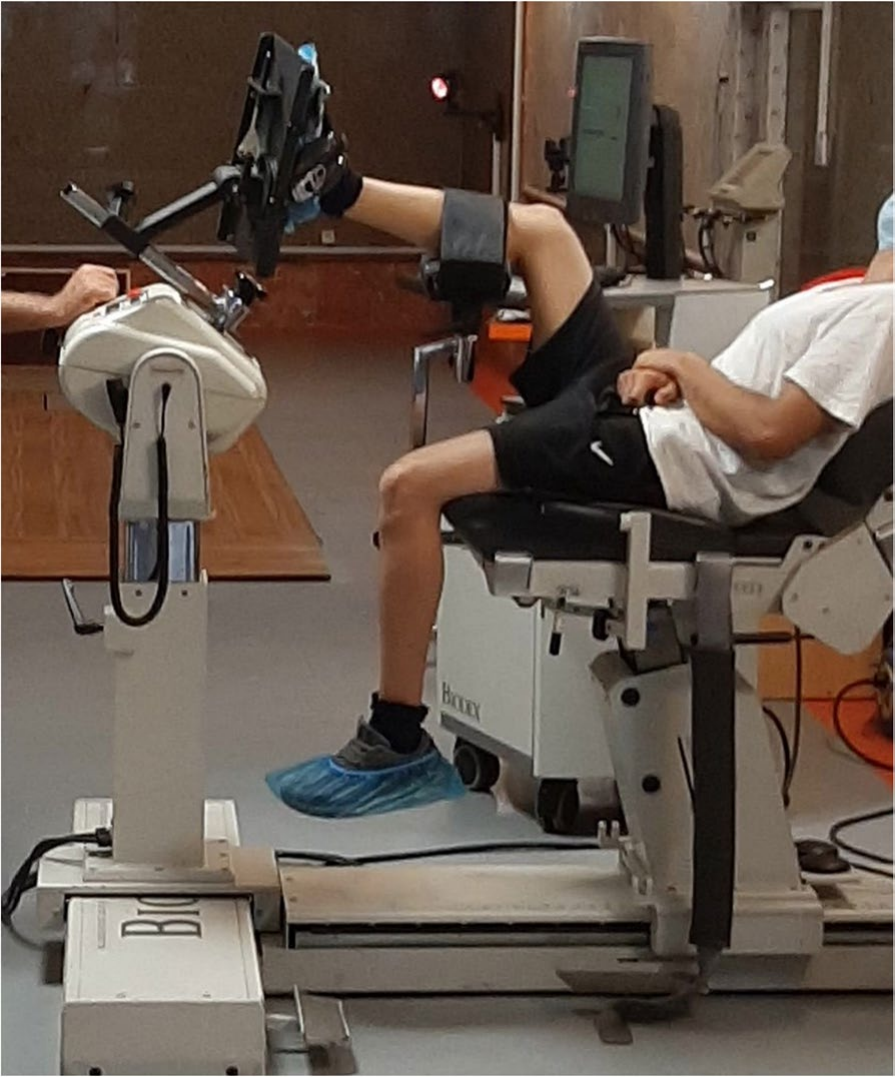
Positioning of the subject for testing active and passive joint-position sense on the Biodex 2 isokinetic dynamometer

Proprioception of joint was measured using a Biodex 2 isokinetic dynamometer (Biodex Medical Systems Inc., Shirley, NY). The subjects lay in supine position on the associated chair and their feet were placed on a special plate (Fig. 1). To prevent extra movement between the sole of the foot and the dynamometer plate, as well as extra knee movement, the subject's foot was fastened to a certain plate with several tight straps. The knee of the tested leg was flexed at 45 degrees and the ankle bent at 15-degree plantar flexion. The subjects' ankles were evaluated in the replication of 15-degree inversion angle and Maximum inversion angle minus 5-degrees. The subjects' eyes were closed with a blindfold during the test. In the passive state, the subjects’ ankle was first taken to maximum eversion by the examiner, their ankle was taken to one of the two test positions and held for 10 s and the subjects were asked to press the stop button when feeling to reach the mentioned position. Their ankle was brought back to eversion and returned to inversion at a rate of 5-degrees per second, and the subject was requested to press the stop button when feeling to reach the mentioned position. The active position is the same as the passive position, except that after the subject's ankle was placed in the test position and returned to eversion, the person was asked to actively bring the ankle to the same position and press the stop button when feeling that the joint angle was replicated. Each situation was repeated twice and the mean of two replications was used for analysis [26, 27].

#### Single-leg jump landing

To perform single-leg jump landing task, the subjects were asked to throw their weight on the non-involved leg on a 40-cm platform and to stand in such a way that the involved leg was bent at knee and held in a relaxed position with hands on hip joint. The subject was then requested to jump up at the command of test taker and land on the center of force plate with the involved leg, maintain balance for 3 s and end the performance by announcement of test taker. Also, before the main test, the subjects were trained to perform the correct jump landing. After learning the jump landing technique, each subject made 3 correct attempts with an interval of 30 s. The correct jump landing technique was checked by the tester, and the test was repeated if the balance of the non-involved leg was disturbed or collided with the ground [28]. Information on the ground reaction force during jump landing was recorded using a force plate device (Kistler, Winterthor, Switzerland, 1000 Hz). The kinetic data were recorded at a sampling frequency of 1000 Hz and filtered using a four-speed downward Butterworth filter and a cut-off frequency of 20. Then, the components of maximum ground reaction forces were normalized via dividing by the weight of subjects and expressed as multiplication of body weight. The mean of data from 3 successful jump landings was used to calculate the variables. The variables calculated in this study were anteroposterior time to stabilization (AP-TTS), mediolateral time to stabilization (ML-TTS), resultant vector of time to stabilization (RV-TTS), mediolateral center of pressure displacement (ML-COP) and anteroposterior center of pressure displacement (AP-COP^) ^ [29].

#### Y-Balance Test (YBT)

Dynamic balance was measured using YBT, which calculates reaching out in individuals in three directions: anterior, posteromedial and posterolateral. Participants were placed in the center of three intersecting lines with the involved foot, then the opposite foot reached in the specified directions as far as it could and returned to the starting point without losing balance. At first, they performed the test one to four times experimentally, and after 5 min of rest, the test was performed three more times in each direction with 15 s of rest between each direction. The maximum distance reached was measured and recorded in three directions: anterior, posteromedial and posterolateral in the last three replications. Afterward, mean of maximum reaching out in all three directions was calculated and divided by the length of person's leg, multiplied by 100 and applied as the dynamic balance score of the person [30].

#### Balance Error Scoring System (BESS)

BESS is a reliable and inexpensive tool for measuring static balance in people with CAI. The participants stood in three positions, each on two different surfaces (stiff and foam), with bare feet and closed eyes for 20 s as follows: 1) On both legs; 2) On a single leg; 3) On legs tandem stance. The grading method was based on the score of error table in six tests. The errors included lifting the arms off the hip, walking, opening the eyes, grabbing something, falling, stepping out of the test position after 5 s, flexion or abduction of thigh more than 30 degrees, and lifting the heel or anterior part of leg off the surface. The total errors of individuals in six tests were recorded as their score with maximum total score in each test being equal to 10. If several errors were committed simultaneously, they were counted as a single error. People who could not stay in the test position for at least 5 s received a score of 10. One-legged standing should be on the non-dominant foot and standing with legs in a row should be on the dominant leg in the front. The tests were done in random order [31].

### Statistical analysis

One-way Analysis of covariance (ANCOVA) (3 × 2, group x time) was used to compare between groups, the pre-test used as the covariate. The follow-up Bonferroni post hoc tests were conducted to identify the difference between groups in the dependent variables. In addition, the paired sample t-test was used to compare the within-group (pre-test to post-test). All analyzes of the present study were performed using SPSS software version 22 at a significance level of α ≥ 0.05.

## Results

**Fig. 2:**
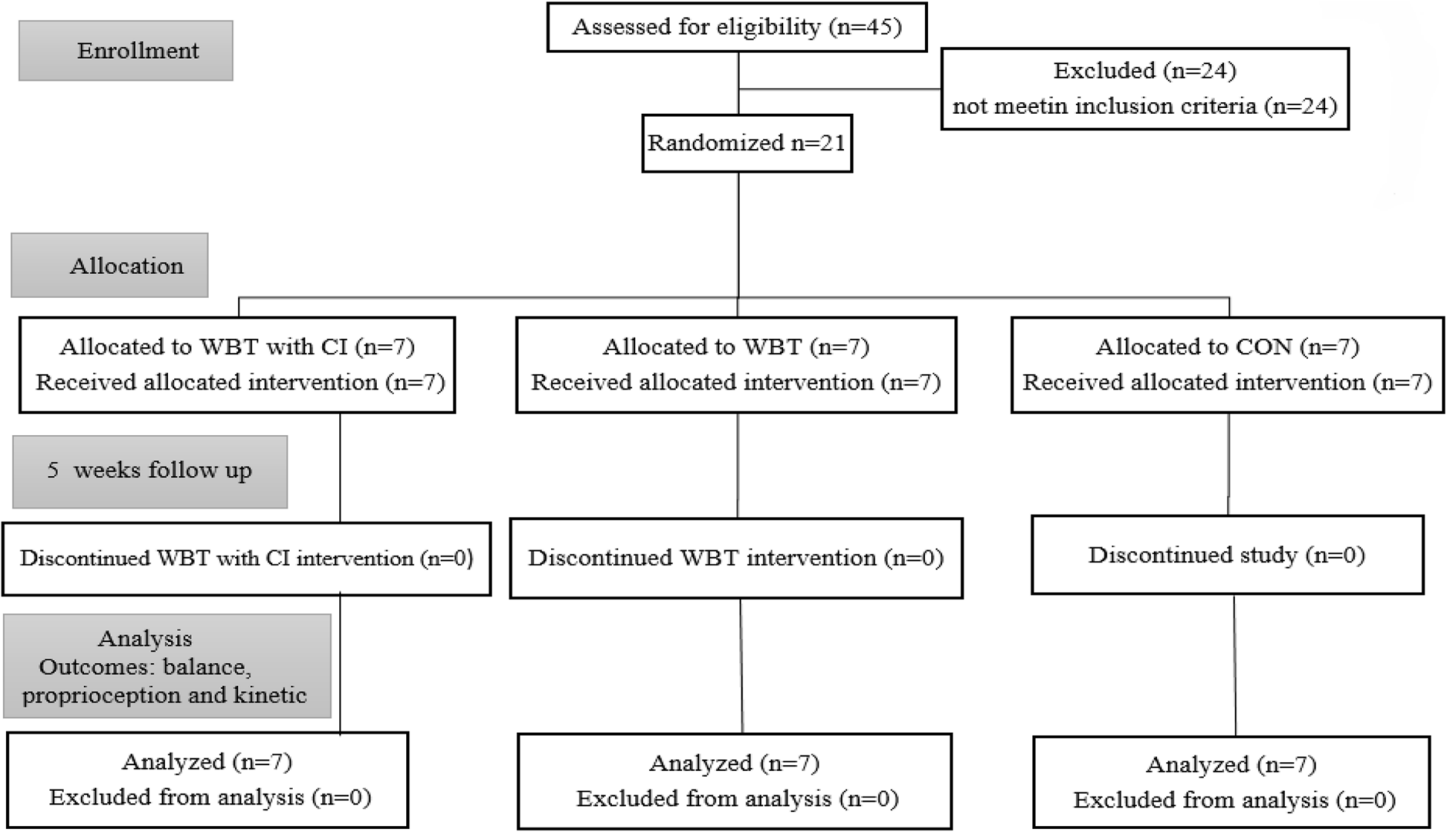
The CONSORT flow diagram. WBT with CI = Wobble Board Training Group with cognitive intervention; WBT = Wobble Board Training Group; CON = Control Group

Out of 45 participants, 24 of them were excluded due to exclusion criteria. The remaining 21 individuals were randomly assigned to three groups of WBT with CI (*n* = 7), WBT without CI (*n* = 7) and control (*n* = 7). During the 5-week intervention, none of the 21 subjects dropped out of the study, and the study ended with the same 21 subjects (Fig. 2). The demographic characteristics of the subjects by groups are listed in Table 1.

This study includes three groups: WBT with CI; WBT without CI; and CON group. As reported in Table 1, in total, the subjects who participated in this study had mean age of 22.66 ± 1.98 years, height of 176.19 ± 5.75, weight of 74.57 ± 4.01 kg, and BMI of 22.06 ± 0.86. To check the homogeneity of subjects’ demographic information in groups, one-way ANOVA test was used. Considering the fact that the value of measured alpha in all variables is > 0.05, we conclude that the three research groups are homogeneous in variables of age, height, weight, and body mass index.

### Comparison of ankle proprioception among groups

Following the intervention period, ANCOVA analysis revealed a main group effect for active replication of 15-degree inversion angle (*F*_(2,18)_ = 25.72*, p* = 0.001*, η*^*2*^ = 0.75,), passive replication of 15-degree inversion angle (*F*_(2,18)_ = 33.66*, p* = 0.001*, η*^*2*^ = 0.79), active replication of maximum inversion angle minus 5-degrees (*F*_(2,18)_ = 20.47*, p* = 0.001*, η*^*2*^ = 0.70,) and passive replication of maximum inversion angle minus 5-degrees (*F*_(2,18)_ = 9.16*, p* = 0.002*, η*^*2*^ = 0.51) (Table 2).

**Table 2:** Ankle proprioception (mean ± SD) and mean difference (95% CI)

	Pre-test	Post-test	Mean Difference (95% CI)	Pre-test	Post-test	Mean Difference (95% CI)
	Active replication of 15-degree inversion angle (Degree)			Passive replication of 15-degree inversion angle (Degree)		
WBT with CI	5.37 ± 1.15	2.97 ± 0.58	-2.40 (-3.2, -1.5) ^a^	5.31 ± 1.46	3.07 ± 0.77	-2.24 (-3.0, -1.4) ^a^
WBT	5.51 ± 1.84	3.17 ± 1.07	-2.34 (-3.2, -1.4) ^a^	4.68 ± 1.04	3.15 ± 0.52	-1.52 (-2.1, -0.8) ^a^
CON	5.30 ± 1.13	4.82 ± 1.08	-0.47 (-1.0, 0.05)	5.07 ± 1.64	4.81 ± 1.12	-0.25 (-1.0, 0.5)
	Active replication of maximum inversion angle minus 5-degrees (Degree)			Passive replication of maximum inversion angle minus 5-degrees (Degree)		
WBT with CI	6.34 ± 1.16	3.27 ± 1.17	-3.07 (-3.5, -2.5) ^a^	5.80 ± 1.99	3.37 ± 1.87	-2.42 (-3.5, -1.3) ^a^
WBT	5.91 ± 0.99	3.21 ± 0.73	-2.70 (-3.8, -1.5) ^a^	6.01 ± 1.72	4.02 ± 1.47	-1.98 (-2.7, -1.2) ^a^
CON	6.11 ± 1.69	5.52 ± 1.10	-0.58 (-1.5, 0.3)	5.90 ± 1.92	6.05 ± 1.92	0.15 (-1.4, 1.7)

*Note*: *WBT with CI *Wobble Board Training Group with cognitive intervention, *WBT *Wobble Board Training Group, *CON *Control Group

^a^ Significant difference compared to CON (*p* < 0.05)

### Comparison of ankle proprioception between pre- and post-test for each group

For the replication of 15-degree inversion angle, the WBT with CI group exhibited a significant difference by -44.69% for active replication (*t* = -6.74, *p* = 0.001) and -42.18% for passive replication (*t* = -6.94, *p* = 0.001). WBT group revealed a significant difference by -35.02% for active replication (*t* = -6.53, *p* = 0.001) and by -32.69% for passive replication (*t* = -5.90, *p* = 0.001). The CON group did not show any changes in active or passive replication (*p* > 0.05) (Table 2).

For the replication of maximum inversion angle minus 5-degrees, WBT with CI group exhibited a significant difference by -48.42% for active replication (*t* = -14.94, *p* = 0.001) and -41.89% for passive replication (*t* = -5.38, *p* = 0.002). WBT group revealed a significant difference by -45.68% for active replication (*t* = -5.64, *p* = 0.001) and by –33.11% for passive replication (*t* = -6.47, *p* = 0.001). The CON group did not show any changes in active or passive replication (*p* > 0.05). In the following, the results of Bonferroni post hoc test have shown that in the post-test, there is a significant difference in all four proprioceptive variables between the CON group with WBT with CI group and the CON group with WBT group (*p* < 0.05). But, no significant difference has been observed between WBT with CI group and WBT group in any of the variables (*p* > 0.05). However, the effect size comparison showed that WBT with CI group led to more improvement than WBT group (Table 2).

### Comparison of jump landing kinetic variables among groups

Following the intervention period, ANCOVA analysis revealed a main group effect for ML-TTS (*F*_(2,18)_ = 19.48*, p* = 0.001*, η*^*2*^ = 0.69,), AP-TTS (*F*_(2,18)_ = 16.46*, p* = 0.001*, η*^*2*^ = 0.66,), RV-TTS (*F*_(2,18)_ = 52.16*, p* = 0.001*, η*^*2*^ = 0.86,), ML-COP (*F*_(2,18)_ = 31.33*, p* = 0.001*, η*^*2*^ = 0.78,) and AP-COP (*F*_(2,18)_ = 30.72*, p* = 0.001*, η*^*2*^ = 0.78) (Table 3).

**Table 3:** Jump landing kinetic variables (mean ± SD) and mean difference (95% CI)

	Pre-test	Post-test	Mean Difference (95% CI)	Pre-test	Post-test	Mean Difference (95% CI)
	Mediolateral Center of pressure (Cm)			Anteroposterior Center of pressure (Cm)		
WBT with CI	2.35 ± 0.28	2.02 ± 0.18	-0.32 (-0.4, -0.1) ^a^	2.30 ± 0.18	2.06 ± 0.13	-0.23 (-0.3, -0.1) ^a^
WBT	2.30 ± 0.33	2.04 ± 0.28	-0.26 (-0.3, -0.1) ^a^	2.22 ± 0.21	2.01 ± 0.14	-0.20 (-0.2, -0.1) ^a^
CON	2.38 ± 0.29	2.38 ± 0.26	-0.001 (-0.05, 0.04)	2.23 ± 0.19	2.25 ± 0.18	-0.02 (-0.002, 0.04)
	Mediolateral time to stabilization (Second)			Anteroposterior time to stabilization (Second)		
WBT with CI	3.10 ± 0.93	2.57 ± 0.67	-0.52 (-0.8, -0.1) ^a^	3.22 ± 0.29	2.92 ± 0.13	-0.30 (-0.5, -0.08) ^a^
WBT	3.23 ± 0.70	2.71 ± 0.61	-0.52 (-0.7, -0.3) ^a^	3.18 ± 0.42	2.88 ± 0.31	-0.29 (-0.4, -0.1) ^a^
CON	3.38 ± 0.32	3.35 ± 0.30	-0.02 (-0.06, -0.01)	3.30 ± 0.25	3.29 ± 0.24	-0.01(-0.04, 0.02)
	Resultant vector time to stabilization (Second)					
WBT with CI	4.52 ± 0.69	3.92 ± 0.45	-0.59 (-0.8, -0.3) ^a^			
WBT	4.57 ± 0.53	4.02 ± 0.39	-0.55 (-0.7, -0.3) ^a^			
CON	4.73 ± 0.28	4.70 ± 0.28	-0.02 (0.01, -1.4)			

*Note*: *WBT with CI *Wobble Board Training Group with cognitive intervention, *WBT *Wobble Board Training Group; CON = Control Group

^a^ Significant difference compared to CON (*p* < 0.05)

### Comparison of jump landing kinetic variables between pre- and post-test for each group

For displacement of COP**,** WBT with CI group exhibited a significant difference by -14.04% for mediolateral (*t* = -6.15, *p* = 0.001) and -10.43% for anteroposterior (*t* = -5.40, *p* = 0.002) displacement. WBT group revealed a significant difference by -11.30% for mediolateral (*t* = -6.42, *p* = 0.001) and by –9.45% for anteroposterior (*t* = -6.33, *p* = 0.001) displacement. The CON group did not show any changes in displacement of COP variables (*p* > 0.05) (Table 3).

For the TTS, the WBT with CI group exhibited a significant difference by -17.09% for mediolateral (*t* = -3.66, *p* = 0.010), -9.31% for anteroposterior (*t* = -3.42, *p* = 0.014) and -13.27% for resultant vector (*t* = -5.43, *p* = 0.002). WBT group revealed a significant difference by -16.09% for mediolateral (*t* = -6.49, *p* = 0.001), –9.43% for anterposterior (*t* = -4.96, *p* = 0.003) and -12.03% for the resultant vector (*t* = -6.83, *p* = 0.001). The CON group did not show any changes in TTS variables (*p* > 0.05). In the following, the results of Bonferroni post hoc test have shown that in the post-test, there is a significant difference in all five kinetic variables between the CON group with WBT with CI group and the CON group with WBT group (*p* < 0.05). But, no significant difference has been observed between WBT with CI group and WBT group in any of the variables (*p* > 0.05). However, the effect size comparison showed that WBT with CI group led to more improvement than WBT group (Table 3).

### Comparison of dynamic and static balance among groups

Following the intervention period, ANCOVA analysis revealed a main group effect for dynamic balance (*F*_(2,18)_ = 6.09*, p* = 0.010*, η*^*2*^ = 0.41,) and static balance (*F*_(2,18)_ = 54.68*, p* = 0.001*, η*^*2*^ = 0.86) (Table 4).

**Table 4:** Dynamic and Static balance (mean ± SD) and mean difference (95% CI)

	Dynamic balance (Normalized to limb length × 100)			Static balance (Number of errors)		
	Pre-test	Post-test	Mean Difference (95% CI)	Pre-test	Post-test	Mean Difference (95% CI)
WBT with CI	71.57 ± 5.38	76.57 ± 4.89	5.00 (0.1, 9.8) ^a^	19.24 ± 4.11	8.71 ± 1.70	-10.71 (-14.0, -7.3) ^a^
WBT	69.42 ± 6.39	74.43 ± 3.86	5.00 (0.6, 9.3) ^a^	17.00 ± 4.16	10.14 ± 1.77	-6.85 (-9.2, -4.4) ^a^
CON	68.28 ± 3.35	68.57 ± 4.39	0.28 (-1.0, 1.6)	18.42 ± 3.59	17.14 ± 2.85	-1.28 (-3.5, 1.0)

*Note*: *WBT with CI *Wobble Board Training Group with cognitive intervention, *WBT *Wobble Board Training Group, *CON *Control Group

^a^ significant difference compared to CON (*p* < 0.05)

### Comparison of dynamic and static balance between pre- and post-test for each group

For dynamic and static balance, WBT with CI group exhibited a significant difference by 6.98% for dynamic (*t* = 2.53, *p* = 0.045) and -54.72% for static (*t* = -7.89, *p* = 0.001) balance. WBT group revealed a significant difference by 7.21% for the dynamic (*t* = 2.82, *p* = 0.030) and by -40.35% for static (*t* = -6.95, *p* = 0.001) balance. The CON group did not show any changes in dynamic nor static balance (*p* > 0.05). In the following, the results of Bonferroni post hoc test have shown that in the post-test, there is a significant difference in dynamic and static balance variables between the CON group with WBT with CI group and the CON group with WBT group (*p* < 0.05). But, no significant difference has been observed between WBT with CI group and WBT group in dynamic and static balance variables (*p* > 0.05). However, the effect size comparison in static balance showed that WBT with CI group led to more improvement than WBT group (Table 4).

## Discussion

The present study was conducted to investigate and compare the effect of a WBT course with and without CI on balance, ankle proprioception and some jump-landing kinetic parameters in active men with CAI. The results showed that a course of WBT with and without CI leads to significant improvement in balance, proprioception and jump-landing kinetic parameters in active men with CAI.

The present study showed that WBT with and without CI reduce mean replication error of 15-degree inversion angle and Maximum inversion angle minus 5-degrees of ankle in both active and passive states in men with CAI after 5 weeks. However, no significant difference was observed between the two experimental groups of WBT with and without CI. The findings of the present study are in line with those of Eils et al. (2010) who stated that neuromuscular training and balance board training can improve ankle proprioception in both inversion and eversion angles [24].

Impaired proprioception is among the performance disabilities associated with CAI. Overall, the proprioception system helps control movements in humans by obtaining information from the environment. After ankle sprain, neuromuscular function decreases due to damage to receptors of ankle ligaments and peroneal nerve [26]. One of the mechanisms and risk factors of external ankle sprain is excessive inversion where people with CAI are defective in recognizing it prior to ground contact. Balance board exercises are among the most common methods in rehabilitation of CAI that have been designed to help retrain the sensory system via improving the function of articular mechanical receptors and restoring the neuromuscular feedback loop [32].

In this regard, Anguish et al. (2018) reported that four weeks of balance training improves the proprioception of people with CAI in inversion, eversion, dorsiflexion, and plantar flexion [33]. Lee et al. (2008) in a research examining the effect of 12 weeks of WBT on ankle balance and proprioception in those with functional ankle instability. The results of this study showed that this training protocol has a significant effect on balance and proprioception in persons with performance instability of the ankle and that the subjects had better scores in proprioception relative to pre-test after 12 weeks of WBT [34]. It has also been reported that healthy people who perform proprioception exercises have 35% lower chance of ankle sprains than those who do not and that these exercises can reduce the risk of re-injury by up to 36% in individuals having a history of ankle sprains [35].

In contrast, Bernier et al. (1998) reported that 6 weeks of balance training and wobble board coordination in individuals with functional ankle instability did not improve active or passive ankle position replication [36]. Riemann et al. (2003) also stated that 4 weeks of coordination and balance exercises with wobble board would not have a significant positive effect on proprioception and balance of healthy active people [37]. However, this difference in the mentioned results could be due to variation in health status of participants (healthy or injured) in the study because Verhagen et al. (2005) have reported that diverse research results may be a function of differences in the degree of defect. For example, one person might experience severe imbalance after ankle sprains, while another one might be subject to defective proprioception [38, 39].

Despite observing a significant improvement of the two experimental groups in ankle proprioception relative to CON group, no significant difference was detected between the two groups of WBT with and without CI. In spite of reviewing previous investigations, the researcher could not find a study examining the effect of balance exercises with CI on ankle proprioception up to this date.

Also, the results of the present study showed that 5 weeks of WBT with and without CI had a significant effect and improved the kinetic parameters of ankle during single-leg jump landing in those with CAI in two experimental groups compared to CON group; however, no significant difference was observed between the two experimental groups. Selected kinetic parameters of this study include ML-TTS, AP-TTS, RV-TTS, ML-COP and AP-COP.

Posture fluctuations are among the parameters that can be examined after ankle sprain. It is a common method to study posture fluctuations by calculating the mean displacement of the leg pressure center. Various studies have presented balance exercises, especially WBT, for post lateral ankle sprain rehabilitation and have also been shown to exert positive effects on reducing posture fluctuations [25, 34, 40–42].

In this study, the height of wobble board from the ground increased by half an inch at each stage. Increase in height increases the inclination angle of wobble board. As a result of this increase in inclination angle of wobble board, the activation rate of leg muscles also increases. WBT are also a type of closed-chain movement exercise that further stimulate the mechanical receptors in muscle joint and sole of the foot [34]. The results of the present study are consistent with those of Lee et al. [34] and Zemkova et al. [40]. Lee et al. also observed a significant reduction in posture fluctuations in both internal–external and anterior–posterior directions after 12 weeks of balance board exercises in individuals with functional ankle instability [34]. Zemkova et al. have reported that six weeks of combined agility and balance exercises with wobble board have a remarkable effect in reducing pressure center fluctuations [40].

On the other hand, the results of the present study are not consistent with findings of Chong et al. [43] and Verhagen et al. [38]. Chong et al. stated that after four weeks of balance training, no significant reduction is observed in pressure center fluctuations [43]. In another study, Verhagen et al. compared the effect of 5 weeks of balance training on fluctuations in the COP while standing on one leg in people with and without a history of lateral ankle sprain, and finally reported that 5 weeks of balance training had no significant effect in reducing fluctuations. The author stated that this lack of significant reduction could be because of the selection of subjects from different sports since athletes in various sports have diverse levels of physical factors such as balance, proprioception or strength. Therefore, depending on the subject's sport, lateral ankle sprain in one person may be associated with an apparent defect in balance and in another person with an obvious defect in strength.

Jump landing is a common movement in sports activities that requires strong dynamic stabilization, and it is also a frequent mechanism for ankle lateral sprain due to excessive ankle inversion. In general, the ability to achieve stabilization more quickly is a positive feature and reduces the risk of injury. Numerous researchers have examined the defect in TTS after repeated lateral ankle sprain and stated that TTS defect is present in both anteroposterior and mediolateral directions, but this defect is more prevalent in anteroposterior direction [44]. Results of the present study showed that five weeks of WBT with and without CI led to a significant reduction in AP-TTS and ML- TTS as well as RV-TTS compared to the CON group. Nevertheless, no significant difference was observed between the two experimental groups in this regard. The findings of the present study are in line with Ross et al. [41] and Mckeon et al. [42].

Ross et al. (2006) reported that coordination exercises with wobble board reduced AP-TTS after two weeks and ML-TTS after four weeks. These findings indicate that achieving stabilization in posteromedial direction requires a longer rehabilitation program [41]. Mckeon et al. (2008) also reported that four weeks of balance training in people with CAI improves performance and postural stability in anteroposterior and posterolateral directions [42].

Melzer et al. (2001) have shown that posture fluctuations increase when performing dual tasks [45]. Also, Rahnama et al. (2010) reported that postural fluctuations in people with ankle instability in both anteroposterior and posteromedial directions in single-leg standing together with CI are higher than single-legged standing without CI [46]. While Shiravi et al. (2016) in a study investigated the effect of dual task on postural fluctuations while standing on one leg in people with CAI and showed that postural fluctuations were decreased in both anteroposterior and posteromedial directions in dual task mode [25].

People with CAI use hip strategy to compensate for the imbalance. This strategy is most likely because they are more confident in maintaining their balance by changing the hip joint instead of the ankle. In other words, to maintain joint stability, they try to lock the ankle joint and reduce the range of joint movement. An error in static balance test was the separation of leg from the surface and walking. After five weeks of balance training, it was observed that the subjects' errors in balance test were decreased relative to pre-test. WBT help people rely more on ankle strategy to have more stabilization in the ankle area, to have more contact with the ground without fear of ankle rotation and to have more contact with ground using ankle movements and range to achieve a better balance [17].

The WBT protocol instructs subjects to hold or rotate the edges of wobble board near the ground gently and in a controlled manner, gradually building up ankle range and not allowing for an excessive or uncontrolled rotation, and this ability to control the movements and stabilization of ankle is itself one of the main factors of maintaining balance and subsequently preventing excessive inversion of the ankle and sprain of lateral ankle ligament.

The results of the present study are consistent with the findings of Lee et al. [34], Wright et al. [3] and Linens et al. [17]. In a study of people with functional ankle instability, Lee et al. (2008) found that subjects scored better on postural control and proprioception after 12 weeks of WBT relative to pre-test [34]. Wright et al. (2017) selected two common rehabilitation methods, including balance board exercises using wobble board and resistance training by stretch. After 12 training sessions over 4 weeks, the research findings showed that balance exercises using wobble board are more effective in rehabilitating CAI than resistance training [3]. Linens et al. (2016) studied the effect of 4 weeks of WBT on balance and performance in people with CAI. The results of this article indicated that four weeks of WBT had a significant positive effect on static and dynamic balance variables and performance of people with CAI, which is consistent with the findings of the present study [17].

Significant improvements in YBT were also observed in the two experimental groups compared to the CON group. Wobble board and YBT are known as two partner tasks with the difference that the wobble board has a moving support surface and individuals must maintain their mass center within this stable range while performing WBT, but YBT has a fixed support surface, the mass center is mobile and persons try to keep their mass centers within a stable range. Thus, it seems that moving support surfaces such as wobble board improve balance on fixed surfaces such as YBT [17].

The results of the present study did not show a significant difference between the two experimental groups, however, WBT with CI showed better scores in post-test static and dynamic balance compared to WBT which is confirmed by Hiyamizu et al. research (2012). In this study, it was shown that adding a cognitive task to a balance exercise did not improve balance in the dual task group compared to the balance exercise group, and no significant difference in balance was observed between the two groups [47]. Furthermore, Melzer et al. (2001) evaluated the effect of cognitive task during a motor task on postural fluctuations and reported that performing a cognitive task during a motor task increases postural fluctuations and decreases balance in individuals [45]. These results are consistent with the theory of limited capacity of attention, which states that when dual tasks are performed simultaneously, if the total attention capacity of the individual is less than the attention required to perform both tasks, the result will be a malfunction of both tasks or one of them, and a decrease in balance while performing a dual task can be a function of this interference theory. In contrast, Shiravi et al. (2016) state that dual cognitive task combined with a motor task leads to a reduction in postural fluctuations [25], which is in line with the results of Andersson et al. (2002) [48]. This discrepancy between research results can be due to differences in inclusion criteria of the subjects, including their age or health status, or because of the type of cognitive task selected in terms of severity or ease.

Because postural control in daily activities, as well as sports and leisure activities and team games usually requires at least another simultaneous task (for example, maintaining balance when thinking about teammates' performance and placement), an athlete should be able to devote some attention to other activities without compromising motor function and postural control. Researchers have also shown that interventions that shift focus away from postural control are more effective in balance training than interventions focusing on postural control [46]. Therefore, it is better that exercise protocols for people with CAI are designed as dual tasks, and each motor activity should be accompanied with a cognitive activity so that the person learns to control posture automatically and without the need for attention.

It should be noted that due to the coronavirus epidemics and the need to comply with health protocols in the laboratory, training clubs and testing facilities, it was not possible to include more subjects in this study. Besides, this research was performed on active men aged 18–25 years with CAI. Therefore, it cannot be generalized to the whole community. On the other hand, considering that the subjects of the present study were active people, it is recommended to recruit subjects who are active in different sports in future research. Moreover, the lack of significant effect of WBT with CI compared to these exercises without CI can be due to the short training period. Therefore, in future research, it is recommended to study the effect of the training protocol mentioned in this study with a training period of more than 5 weeks. It is also suggested that in a similar study, the variables of this study be compared and evaluated between injured and healthy feet in people with CAI.

## Conclusions

Therefore, according to the findings of the present study, it can be concluded that a course of WBT with and without CI leads to a significant improvement in balance, proprioception and jump-landing kinetic parameters, which include the TTS and displacement of COP in active men with CAI. Moreover, according to the effect size calculated for each variable, it was found that after five weeks of training, the WBT group with CI generally led to further improvement relative to WBT group without CI; however, this difference was not significant in any of the variables.

## Data Availability

The datasets used and analysed during the current study are available from the corresponding author on reasonable request.

## Abbreviations

WBT: Wobble board training
CI: Cognitive intervention
CAI: Chronic ankle instability
COP: Center of pressure
FADI: Foot & Ankle Disability Index
FADI-S: Foot & Ankle Disability Index-Sports
CON: Control
AP-TTS: Anteroposterior time to stabilization
ML-TTS: Mediolateral time to stabilization
RV-TTS: Resultant vector of time to stabilization
ML-COP: Mediolateral center of pressure displacement
AP-COP: Anteroposterior center of pressure displacement
YBT: Y-Balance Test
BESS: Balance Error Scoring System

## References

[1] Wright CJ, Linens SW (2017). Patient-reported efficacy 6 months after a 4-Week rehabilitation intervention in individuals with chronic ankle instability. J Sport Rehabil.

[2] Fusco A, Giancotti GF, Fuchs PX, Wagner H, Varalda C, Cortis C (2019). Wobble board balance assessment in subjects with chronic ankle instability. Gait Posture.

[3] Wright CJ, Linens SW, Cain MS (2017). A randomized controlled trial comparing rehabilitation efficacy in chronic ankle instability. J Sport Rehabil.

[4] Hertel J (2002). Functional anatomy, pathomechanics, and pathophysiology of lateral ankle instability. J Athl Train.

[5] Frigg A, Magerkurth O, Valderrabano V, Ledermann HP, Hintermann B (2007). The effect of osseous ankle configuration on chronic ankle instability. Br J Sports Med.

[6] Karlsson J, Bergsten T, Lansinger O, Peterson L (1988). Reconstruction of the lateral ligaments of the ankle for chronic lateral instability. J Bone Joint Surg Am.

[7] Tümer N, Vuurberg G, Blankevoort L, Kerkhoffs GMMJ, Tuijthof GJM, Zadpoor AA (2019). Typical Shape Differences in the Subtalar Joint Bones Between Subjects with Chronic Ankle Instability and Controls. J Orthop Res.

[8] Mettler A, Chinn L, Saliba SA, McKeon PO, Hertel J (2015). Balance training and center-of-pressure location in participants with chronic ankle instability. J Athl Train.

[9] Daneshjoo A, Tavakol A, Sadeghi H (2020). Effect of twelve sessions of Tai Chi exercise on static and dynamic balance in young girls. Int J Kinesiol Sport Sci.

[10] Song GB, Park EC (2015). Effect of dual tasks on balance ability in stroke patients. J Phys Ther Sci.

[11] Oxendine JB (1970). Emotional Arousal and Motor Performance. Quest.

[12] Kim AS, Needle AR, Thomas SJ, Higginson CI, Kaminski TW, Swanik CB (2016). A sex comparison of reactive knee stiffness regulation strategies under cognitive loads. Clin Biomech (Bristol, Avon).

[13] Hart HF, Culvenor AG, Collins NJ, Ackland DC, Cowan SM, Machotka Z (2016). Knee kinematics and joint moments during gait following anterior cruciate ligament econstruction: a systematic review and meta-analysis. Br J Sports Med.

[14] Burcal CJ, Needle AR, Custer L, Rosen AB (2019). The effects of cognitive loading on motor behavior in injured individuals: a systematic review. Sports Med.

[15] Silva P de B, Oliveira AS, Mrachacz-Kersting N, Laessoe U, Kersting UG (2016). Strategies for equilibrium maintenance during single leg standing on a wobble board. Gait Posture.

[16] Valle MS, Casabona A, Cavallaro C, Castorina G, Cioni M. Learning upright standing on a multiaxial balance board. PLoS One. 2015;10(11). Available from: https://pubmed.ncbi.nlm.nih.gov/26544694/. [Cited 2021 May 3]. 10.1371/journal.pone.0142423 PMC4636294 26544694

[17] Linens SW, Ross SE, Arnold BL (2016). Wobble board rehabilitation for improving balance in Ankles with chronic instability. Clin J Sport Med.

[18] Canning CG, Ada L, Woodhouse E (2008). Multiple-task walking training in people with mild to moderate Parkinson’s disease: a pilot study. Clin Rehabil.

[19] Silsupadol P, Lugade V, Shumway-Cook A, van Donkelaar P, Chou LS, Mayr U (2009). Training-related changes in dual-task walking performance of elderly persons with balance impairment: a double-blind, randomized controlled trial. Gait Posture.

[20] Bhaskaran K, dos-Santos-Silva I, Leon DA, Douglas IJ, Smeeth L (2018). Association of BMI with overall and cause-specific mortality: a population-based cohort study of 3·6 million adults in the UK. Lancet Diabetes Endocrinol.

[21] Springer S, Gottlieb U. Effects of dual-task and walking speed on gait variability in people with chronic ankle instability: A cross-sectional study. BMC Musculoskelet Disord. 2017;18(1). Available from: https://pubmed.ncbi.nlm.nih.gov/28732483/. [Cited 2021 May 3]. 10.1186/s12891-017-1675-1 PMC5522594 28732483

[22] Gribble PA, Delahunt E, Bleakley CM, Caulfield B, Docherty CL, Fong DTP (2014). Selection criteria for patients with chronic ankle instability in controlled research: a position statement of the international ankle consortium. J Athl Train.

[23] Schultz RA, Miller DC, Kerr CS, Micheli L (1984). Mechanoreceptors in human cruciate ligaments: a histological study. J Bone Jt Surg - Ser A.

[24] Eils E, Schröter R, Schröderr M, Gerss J, Rosenbaum D (2010). Multistation proprioceptive exercise program prevents ankle injuries in basketball. Med Sci Sports Exerc.

[25] Shiravi Z, TalebianMoghadam S, Hadian MR, Olyaei G (2017). Effect of cognitive task on postural control of the patients with chronic ankle instability during single and double leg standing. J Bodyw Mov Ther.

[26] Hagen M, Lemke M, Lahner M (2018). Deficits in subtalar pronation and supination proprioception in subjects with chronic ankle instability. Hum Mov Sci.

[27] Willems T, Witvrouw E, Verstuyft J, Vaes P, De Clercq D (2002). Proprioception and Muscle Strength in Subjects With a History of Ankle Sprains and Chronic Instability. J Athl Train.

[28] Caulfield B, Garrett M (2004). Changes in ground reaction force during jump landing in subjects with functional instability of the ankle joint. Clin Biomech.

[29] Winter DA (2009). Biomechanics and Motor Control of Human Movement. Vol. 7, Biomechanics and Motor Control of Human Movement.

[30] Hall EA, Docherty CL, Simon J, Kingma JJ, Klossner JC (2015). Strength-training protocols to improve deficits in participants with chronic ankle instability: a randomized controlled trial. J Athl Train.

[31] Smith BI, Curtis D, Docherty CL (2018). Effects of hip strengthening on neuromuscular control, hip strength, and self-reported functional deficits in individuals with chronic Ankle instability. J Sport Rehabil.

[32] Rozzi SL, Lephart SM, Sterner R, Kuligowski L (1999). Balance training for persons with functionally unstable ankles. J Orthop Sports Phys Ther.

[33] Anguish B, Sandrey MA (2018). Two 4-week balance-training programs for chronic ankle instability. J Athl Train.

[34] Lee AJY, Lin WH (2008). Twelve-week biomechanical ankle platform system training on postural stability and ankle proprioception in subjects with unilateral functional ankle instability. Clin Biomech.

[35] Han J, Anson J, Waddington G, Adams R, Liu Y. The role of ankle proprioception for balance control in relation to sports performance and injury. Vol. 2015, BioMed Research International. Hindawi Publishing Corporation; 2015. Available from: https://pubmed.ncbi.nlm.nih.gov/26583139/. [Cited 2021 May 4]. 10.1155/2015/842804 PMC4637080 26583139

[36] Bernier JN, Perrin DH (1998). Effect of coordination training on proprioception of the functionally unstable ankle. J Orthop Sports Phys Ther.

[37] Riemann BL, Tray NC, Lephart SM (2003). Unilateral multiaxial coordination training and ankle kinesthesia, muscle strength, and postural control. J Sport Rehabil.

[38] Verhagen E, Bobbert M, Inklaar M, Van Kalken M, Van Der Beek A, Bouter L (2005). The effect of a balance training programme on centre of pressure excursion in one-leg stance. Clin Biomech.

[39] Heishman AD, Peak KM, Daub BD, Miller RM, Freitas EDS (2021). Does footwear influence countermovement jump parameters used to assess performance in collegiate basketball players?. Int J Kinesiol Sport Sci.

[40] Zemková E, Hamar D (2010). The effect of 6-week combined agility-balance training on neuromuscular performance in basketball players. J Sport Med Phys Fit.

[41] Ross SE, Guskiewicz KM (2006). Effect of coordination training with and without stochastic resonance stimulation on dynamic postural stability of subjects with functional ankle instability and subjects with stable ankles. Clin J Sport Med.

[42] Mckeon PO, Ingersoll CD, Kerrigan DC, Saliba E, Bennett BC, Hertel J (2008). Balance training improves function and postural control in those with chronic ankle instability. Med Sci Sports Exerc.

[43] Chong RKY, Ambrose A, Carzoli J, Hardison L, Jacobson B (2001). Source of improvement in balance control after a training program for ankle proprioception. Percept Mot Skills.

[44] Wright CJ, Arnold BL, Ross SE (2016). Altered kinematics and time to stabilization during drop-jump landings in individuals with or without functional ankle instability. J Athl Train.

[45] Melzer I, Benjuya N, Kaplanski J (2001). Age-related changes of postural control: Effect of cognitive tasks. Gerontology.

[46] Rahnama L, Salavati M, Akhbari B, Mazaheri M (2010). Attentional demands and postural control in athletes with and without functional ankle instability. J Orthop Sports Phys Ther.

[47] Hiyamizu M, Morioka S, Shomoto K, Shimada T (2012). Effects of dual task balance training on dual task performance in elderly people: A randomized controlled trial. Clin Rehabil.

[48] Andersson G, Hagman J, Talianzadeh R, Svedberg A, Larsen HC (2002). Effect of cognitive load on postural control. Brain Res Bull.

